# Prognostic Significance of Elevated Cardiac Troponin-T Levels in Acute Respiratory Distress Syndrome Patients

**DOI:** 10.1371/journal.pone.0040515

**Published:** 2012-07-12

**Authors:** Matthew B. Rivara, Ednan K. Bajwa, James L. Januzzi, Michelle N. Gong, B. Taylor Thompson, David C. Christiani

**Affiliations:** 1 Department of Medicine, Massachusetts General Hospital, Boston, Massachusetts, United States of America; 2 Pulmonary and Critical Care Unit, Massachusetts General Hospital, Harvard Medical School, Boston, Massachusetts, United States of America; 3 Cardiology Unit, Massachusetts General Hospital, Harvard Medical School, Boston, Massachusetts, United States of America; 4 Critical Care Division, Montefiore Medical Center, Albert Einstein College of Medicine, Bronx, New York, United States of America; 5 Department of Environmental Health, Harvard School of Public Health, Boston, Massachusetts, United States of America; University of Colorado Denver, United States of America

## Abstract

**Background:**

Elevated levels of biochemical markers of myocardial necrosis have been associated with worsened outcomes in Acute Respiratory Distress Syndrome (ARDS), but there are few prospective data on this relationship. We investigated elevated cardiac troponin T (cTnT) levels and their relationship with outcome in patients with ARDS.

**Methods:**

A prospective cohort study of patients with ARDS was conducted at a tertiary-care academic medical center. Patients had blood taken within 48 hours of ARDS onset and assayed for cTnT. Patients were followed for the outcomes of 60-day mortality, number of organ failures, and days free of mechanical ventilation. Echocardiographic and electrocardiographic (ECG) data were analyzed for signs of myocardial ischemia, infarction, or other myocardial dysfunction.

**Results:**

177 patients were enrolled, 70 of whom died (40%). 119 patients had detectable cTnT levels (67%). Median cTnT level was 0.03 ng/mL, IQR 0–0.10 ng/mL, and levels were higher among non-survivors (*P* = .008). Increasing cTnT level was significantly associated with increasing mortality (*P* = .008). The association between increasing cTnT level and mortality remained significant after adjustment in a multivariate model (HR_adj_ = 1.45, 95% CI 1.17–1.81, *P* = .001). Elevated cTnT level was also associated with increased number of organ failures (*P* = .002), decreased number of days free of mechanical ventilation (*P* = .03), echocardiographic wall motion abnormalities (*P* = 0.001), and severity of tricuspid regurgitation (*P* = .04). There was no association between ECG findings of myocardial ischemia or infarction and elevated cTnT.

**Conclusions:**

Elevated cTnT levels are common in patients with ARDS, and are associated with worsened clinical outcomes and certain echocardiographic abnormalities. No association was seen between cTnT levels and ECG evidence of coronary ischemia.

## Introduction

The Acute Respiratory Distress Syndrome (ARDS) is marked by considerable cardiovascular strain. This is related to severe respiratory compromise and hypoxemia caused by endothelial injury and capillary leak, which result in alveolar filling and subsequent respiratory failure [1]. Hypoxemia stimulates increased cardiac output, which can result in myocardial strain in the setting of decreased oxygen supply and pulmonary hypertension due to hypoxic pulmonary vasoconstriction, pulmonary endothelial injury, and *in situ* thrombosis of the pulmonary vasculature [1]. This condition is further exacerbated by pulmonary capillary obliteration and lung fibrosis as the syndrome progresses, as well as by increased intrathoracic pressure from mechanical ventilation [1], [2]. Patients with sepsis and septic shock, which are commonly associated with ARDS, are known to have a syndrome of myocardial dysfunction at least partly due to inflammation-induced myocardial apoptosis [3]. Finally, myocardial dysfunction may be exacerbated by increased myocardial oxygen demand during critical illness, in the setting of preexisting coronary heart disease (CHD) restricting coronary arterial blood flow [4]. In sum, while ARDS is considered a pulmonary disease, the cardiovascular consequences from this devastating illness are substantial.

Echocardiography is frequently used in the setting of critical illness to assess cardiovascular structure and function. Patients with sepsis and septic shock undergo echocardiography to evaluate the etiology of pulmonary edema and ventricular dysfunction, and the sensitivity of transthoracic echocardiography for cardiac causes of shock has been reported to be as high as 100% [5]–[7]. Changes over time in pulmonary artery hemodynamics have been associated with prognosis in critically ill patients with acute severe respiratory failure [8]. Likewise, electrocardiography (ECG) is an important and ubiquitous diagnostic tool in the intensive care unit (ICU), and has been shown to perform reasonably well in detecting myocardial ischemia and infarction in ICU patients [9]. Though it has been demonstrated that elevated levels of cardiac biomarkers are associated with increased mortality and length of stay in ICU populations, the underlying cause of such biomarker elevations remains to be elucidated [10]. No study to date has described the echocardiographic and ECG characteristics of ICU patients with elevated cardiac biomarkers and sepsis or ARDS.

Troponin assays are widely used for their diagnostic and prognostic utility in several clinical settings, including detection or exclusion of myocardial infarction (MI), heart failure, and pulmonary embolism, and as supportive tests in sepsis and stroke [11]–[13]. We have previously shown that elevated levels of blood biochemical markers of myocardial necrosis are associated with increased morbidity and mortality in ARDS patients [14]. However, this analysis was limited by its retrospective nature and subject to selection bias because the decision to test for biomarker levels was made by treating clinicians. In addition, the analysis did not incorporate available echocardiographic and ECG data, which limited our ability to assess for concomitant acute coronary syndromes and/or underlying coronary artery disease as the etiology of abnormal elevations in cardiac biomarkers. Therefore, we sought to evaluate the association between cardiac troponin T (cTnT) levels and outcome prospectively in patients with ARDS, incorporating echocardiographic and ECG data for the study population. We hypothesized that elevated levels of cTnT are associated with worse adjusted outcomes in patients with ARDS above and beyond that informed by more detailed phenotyping using ultrasound or ECG.

## Materials and Methods

### Ethics Statement

All aspects of the study protocol were approved by the Partners Human Research Committee (Protocol #: 1999-P-008607/98). Informed written consent was obtained from all patients if possible, or from surrogates.

### Study Design and Enrollment

Study patients were drawn from a prospective cohort assembled for a molecular epidemiology study of ARDS, and were distinct from those included in our prior study. All adult patients admitted to the intensive care units (ICU) at our institution (Massachusetts General Hospital, Boston, MA) from September 1999 to May 2005 were considered for enrollment if they had at least one risk factor for ARDS from among sepsis, septic shock, pneumonia, trauma, multiple transfusions, or aspiration. Inclusion and exclusion criteria were as previously defined [15]. ARDS was defined as having respiratory failure requiring mechanical ventilation and meeting North American-European Consensus Committee (NAECC) criteria for ARDS [16].

### Data Collection

Demographic data and data used for calculation of an Acute Physiology and Chronic Health Evaluation (APACHE) III score were collected at baseline. Zero was assigned to components of the APACHE score with missing physiology variables according to the methods used by the APACHE III investigators. Patients were screened on each ICU day for presence of ARDS. Data were recorded until ICU discharge or for 28 days if the patient remained in the ICU. Patients were followed for the primary endpoint of 60-day survival. For patients who had been discharged from the hospital prior to 60-days post-enrollment, phone calls were made by study staff members to patients’ homes, rehabilitation facilities, and skilled nursing facilities to ascertain survival status. Secondary endpoints were number of ventilator-free days and average daily multiple organ dysfunction score (MODS), which was calculated as defined by the criteria of Brussels, with one point awarded per organ system failure [16].

Data regarding cardiac history and risk factors were obtained from the history upon admission. Coronary artery disease was considered to be present if reported by patients or their surrogates, documented in the medical record, or if there were confirmatory testing data available. Echocardiographic and ECG data were retrospectively obtained for patients with available data within 48 hours of blood sample collection for cTnT. Patients underwent echocardiographic/ECG testing as part of clinical care when deemed necessary by treating physicians. Left ventricular mass was calculated using the formula proposed by Devereaux [17]. Mitral and tricuspid valvular regurgitation were recorded on a 4 point scale, where 0 represented no regurgitation, 1 was trace, 2 was mild, 3 was moderate, and 4 was severe regurgitation. ECG results were obtained from any ECGs available in the patient’s medical record within 48 hours of blood sample collection for cTnT. As per the method proposed by Lim et al., each ECG was examined by a study physician (MR) for the presence or absence of myocardial ischemia [9]. This was based on the presence or absence of six findings that may represent acute MI or myocardial ischemia as per ESC/ACC guidelines: 1) pathologic Q waves, 2) ST-segment elevation in greater than or equal to two contiguous leads, 3) ST-segment depression in greater than or equal to two contiguous leads, 4) symmetric inversion of T waves of ≥1 mm in greater than or equal to two contiguous leads, 5) T-wave flattening, and 6) left bundle branch block (LBBB) [18]. For quality control purposes, ECG interpretations by the study physician were compared against formal ECG interpretation when available. This approach was used for verification purposes only. If there were important discordances, we had planned to conduct independent verification by a second study physician, but no substantive differences were found. The study physician collecting echocardiogram and ECG data (MR) was blinded to other study variables and outcomes.

### Sample Collection and Testing

Blood samples were taken in EDTA-treated plasma from within 48 hours of fulfillment of all ARDS criteria and stored at a temperature of –80 C until testing. Levels of cTnT were tested using Roche Elecsys 2010 immunoassay analyzers according to manufacturer protocols. Patients were excluded if blood samples could not be obtained, most commonly due to delays in obtaining informed consent. When excluded patients were compared to tested patients, there were no significant differences in terms of age, gender, ethnicity, APACHE III score, or other relevant baseline characteristics.

### Statistical Analysis

Statistical analyses were performed using SAS software, version 9.1 (SAS Institute Incorporated, Cary, North Carolina, USA), as well as with STATA software, version 11.0 (StataCorp, College Station, Texas, USA). Univariate analyses were performed using Chi-squared tests, two-sample t-tests, or Wilcoxon rank-sum tests as appropriate. Log transformation was performed on cTnT levels to achieve normality. To test the effect of increasing cTnT log-quartile on mortality rate, patients were divided into cTnT log-quartiles and the Cochrane-Armitage chi-square test of trend was applied.

Cox proportional hazards modeling was used to further analyze the association between increasing cTnT level (modeled as a continuous variable) and hazard of 60-day mortality. We planned *a priori* to adjust this analysis for age and severity of illness (using APACHE III score modified to remove the age component). Other covariates of clinical relevance or those with significant differences in baseline characteristics were considered for inclusion in this analysis using a backward stepwise selection algorithm with a *P* value of ≤0.2 as the cutoff for inclusion. All demographic, chronic health, etiologic, and physiologic variables with significant differences between groups as listed in Table 1 were considered for inclusion. Two other covariates, history of coronary artery disease and presence of acute hepatic failure, met criteria for inclusion. In view of our prior finding that the association of elevated biomarker levels with mortality is modified by age and severity of illness, we made the *a priori* decision to test for effect modification by these variables by adding interaction terms to the multivariable model.

**Table 1:** Baseline Characteristics of Study Population.

	*Survivors* (n = 107)	Non-survivors (n = 70)	*P*
***Demographics***			
Age (years)	56.0 (42.0–71.0)	71.5 (59.0–80.0)	<.0001
*Female gender*	51 (48%)	28 (40%)	.35
Caucasian race	99 (93%)	66 (94%)	.64
***Acute Illness/ARDS risk factor***			
Pneumonia	79 (75%)	53 (76%)	.86
Septic shock	58 (55%)	46 (66%)	.18
Sepsis (w/o shock)	34 (32%)	19 (27%)	.49
Trauma	6 (6%)	1 (1%)	.16
Aspiration	8 (8%)	8 (11%)	.38
Multiple transfusions	8 (8%)	6 (9%)	.81
Acute hepatic failure	0	4 (5.7%)	.01
***Cardiac History***			
Known coronary artery disease	18 (17%)	26 (37%)	.003
Prior myocardial infarction	12 (12%)	14 (20%)	.12
Prior coronary artery bypass grafting	4 (4%)	6 (9%)	.19
Hyperlipidemia history	20 (19%)	13 (19%)	.91
Hypertension history	41 (39%)	33 (47%)	.31
Congestive heart failure	8 (8%)	10 (14%)	.16
***Chronic Health***			
Cirrhosis	2 (1.9%)	8 (11.4%)	.007
Corticosteroid therapy	14 (13%)	16 (23%)	.10
Diabetes history	25 (23%)	15 (21%)	.74
Smoking history	59 (66%)	40 (65%)	.89
Metastatic solid-organ malignancy	3 (2.8%)	2 (2.8%)	.99
***Baseline physiologic variables***			
APACHE III score	73.0 (59.0–87.0)	90.0 (75.0–105.0)	<.0001
Lowest WBC count (1000/mm^3^)	12.1 (6.7–16.0)	11.6 (7.4–17.4)	.61
Highest WBC count (1000/mm^3^)	16.1 (11.0–20.8)	16.4 (11.3–24.3)	.70
Lowest mean arterial pressure (mm Hg)	58.0 (54.0–63.0)	56.0 (51.0–60.0)	.04
Need for vasopressor therapy	64 (60%)	47 (67%)	.32
Serum BUN (mg/dL)	23.0 (14.0–37.0)	34.0 (20.0–50.0)	.03
Serum creatinine (mg/dL)	1.2 (0.9–1.9)	1.5 (0.8–2.2)	.59
24-hour urine output (cc)	1687.5 (1082–2560)	1214.0 (710–1940)	.008
Serum total bilirubin (mg/dL)	0.7 (0.4–1.1)	0.90 (0.5–2.5)	.01
Red blood cell units transfused	1.5 (0–3.0)	2 (0–4.0)	.16
Platelet count	178.0 (116.0–263.0)	193.5 (80.0–267.0)	.35
Tidal volume (cc per kg of ideal body weight)	9.1 (7.6–10.7)	8.0 (7.0–10.1)	.07
Positive End-Expiratory Pressure (cm H_2_O)	10.0 (6.0–10.0)	8.0 (5.0–12.0)	.43

Continuous variables are presented as median (interquartile range), categorical variables as number of patients in each category and (% of the total category).

For further stratified analyses, patients were divided into groups according to whether cTnT level was above or below a receiver operator characteristic (ROC)-derived cutpoint. ROC curve analysis was conducted and the method of Youden was used to determine the optimal ROC cutpoint for predicting mortality [19]. This cutpoint (0.036 ng/mL) was similar to the consensus cutpoint for diagnosis of acute MI [20]. Kaplan-Meier curves were constructed using cTnT groups as strata and compared using log-rank testing. Daily MODS scores were compared between patients in each cTnT strata using repeated-measures ANOVA. Ventilator-free days were compared between strata using Wilcoxon rank-sum testing.

Echocardiographic and ECG univariate analyses were performed using Chi-squared tests, two-sample t-tests, or Wilcoxon rank-sum tests as appropriate. P values of ≤0.05 were considered statistically significant. For the echocardiographic and ECG analysis, elevated cardiac marker levels were defined according to Universal Definition of Myocardial Infarction cut off strategy of the lowest cut-off providing <10% imprecision, namely a cTnT (Roche Diagnostics, Basel, Switzerland) of ≥0.03 ng/mL.

## Results

### Study Population and cTnT Levels

177 patients were included in the study. Baseline characteristics of the study population, shown in Table 1, are sorted by the primary outcome of 60-day survival. Survivors were significantly younger than non-survivors and had significantly lower severity of illness (APACHE III score). Non-survivors were more likely to have acute hepatic failure or chronic cirrhosis, had significantly lower mean arterial pressure and urine output, and significantly higher serum BUN and bilirubin. Overall mortality rate was 40%. 119 patients had detectable cTnT levels (67%). Median cTnT concentration was 0.03 ng/mL with an interquartile range (IQR) of 0–0.10 ng/mL.

Among factors contributing to cTnT levels, patients with prior coronary artery disease (CAD) had significantly higher cTnT levels than those without CAD, (median 0.08, IQR 0.03–0.30 vs. median 0.02, IQR 0–0.07, *P*<0.0001). In addition, patients with CAD were slightly more likely to have a detectable cTnT level (86% vs. 61%, *P* = .002). Because of the nature of the strong pathophysiologic relationship between CAD and diabetes, we also evaluated whether patients with reported diabetes had elevated cTnT levels despite the absence of known CAD. Among the 18 patients who fell into this subgroup, the proportion of detectable cTnT levels was similar when compared to non-diabetics (67% vs. 60%, *P* = .58).

Patients with septic shock (n = 106) were more likely to have detectable cTnT, but cTnT was still detected in over half of patients without septic shock (72% vs. 55%, *P* = .03). Septic shock patients had significantly higher cTnT levels (median 0.03, IQR 0–0.12 vs. median 0.02, IQR 0–0.06, *P* = .03) than those without septic shock. Among 71 patients without septic shock, only 18 had no clinical evidence or suspicion of infection; of these patients, 11 had detectable cTnT levels, which was not significantly different from the proportion of patients with documented or suspected infection who had detectable cTnT levels (61% vs. 65%, *P* = .80). There was no significant difference in cTnT levels between patients with and without infection (median 0.03, IQR 0–0.1 vs. median 0.03, IQR 0–.06, *P* = .46).

### cTnT and ARDS Outcome

**Figure 1:**
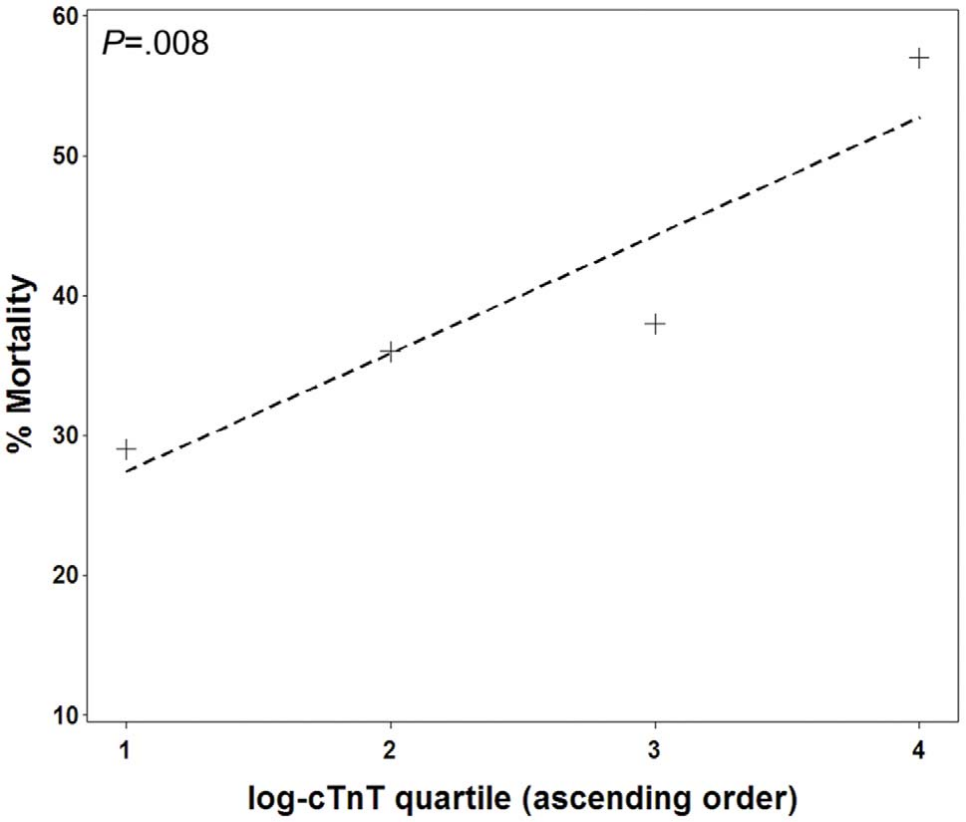
Relationship between cTnT log quartile and mortality, with superimposed trend line. (*P* value stated for Cochran-Armitage test of trend).

ARDS nonsurvivors had significantly higher cTnT levels than survivors (0.042 ng/mL, IQR 0.01–0.21 vs. 0.022, IQR 0–0.08, *P* = .008). Mortality increased monotonically with increasing cTnT log-quartile; this association was statistically significant (Figure 1).

Increasing cTnT level was significantly associated with increased mortality, with hazard ratio (HR) of 1.33 per 1 ng/mL increase (95% CI 1.10–1.62, *P* = 0.003). This association remained significant after adjusting for age, APACHE III score, presence of acute hepatic failure, and presence of CAD in the multivariable model (Table 2). Addition of interaction terms to the model did not show evidence of effect modification by age or APACHE III score.

**Table 2:** Results of Cox proportional hazards model for association of elevated cardiac troponin T level with 60-day Mortality.

*Variable*	*Hazard Ratio*	95% CI	*P*
cTnT (per 1 ng/mL)	1.44	1.14–1.81	.002
Age (per year)	1.04	1.02–1.06	<.0001
APACHE III (per point)	1.02	1.01–1.03	.007
Coronary artery disease	1.39	0.83–2.33	.20
Acute hepatic failure	4.25	1.31–13.75	.02

**Figure 2:**
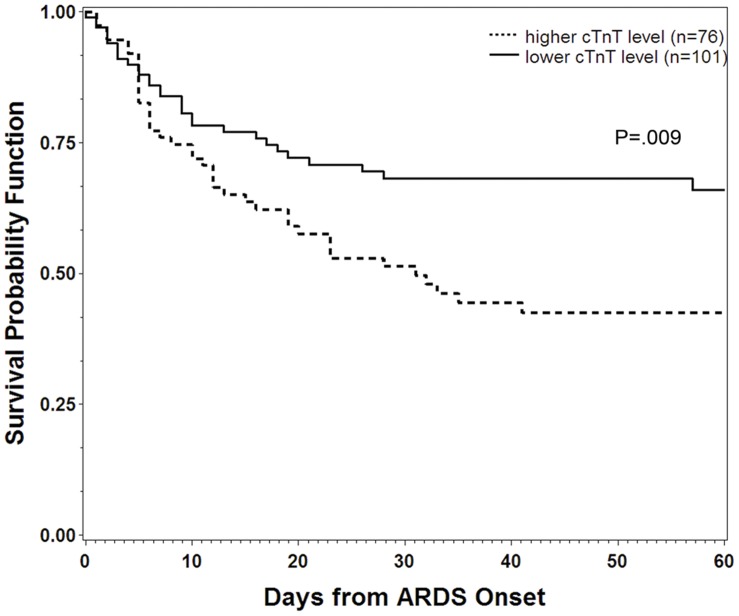
Survival time by cTnT stratum. (Patients stratified according to ROC-determined cutpoint of 0.036 ng/mL, *P* value stated for log-rank test).

Patients were then grouped into strata according to cTnT level above and below 0.036 ng/mL as described. Kaplan-Meier curves demonstrating 60-day mortality in each stratum are shown in Figure 2.

### Secondary Outcomes

**Figure 3:**
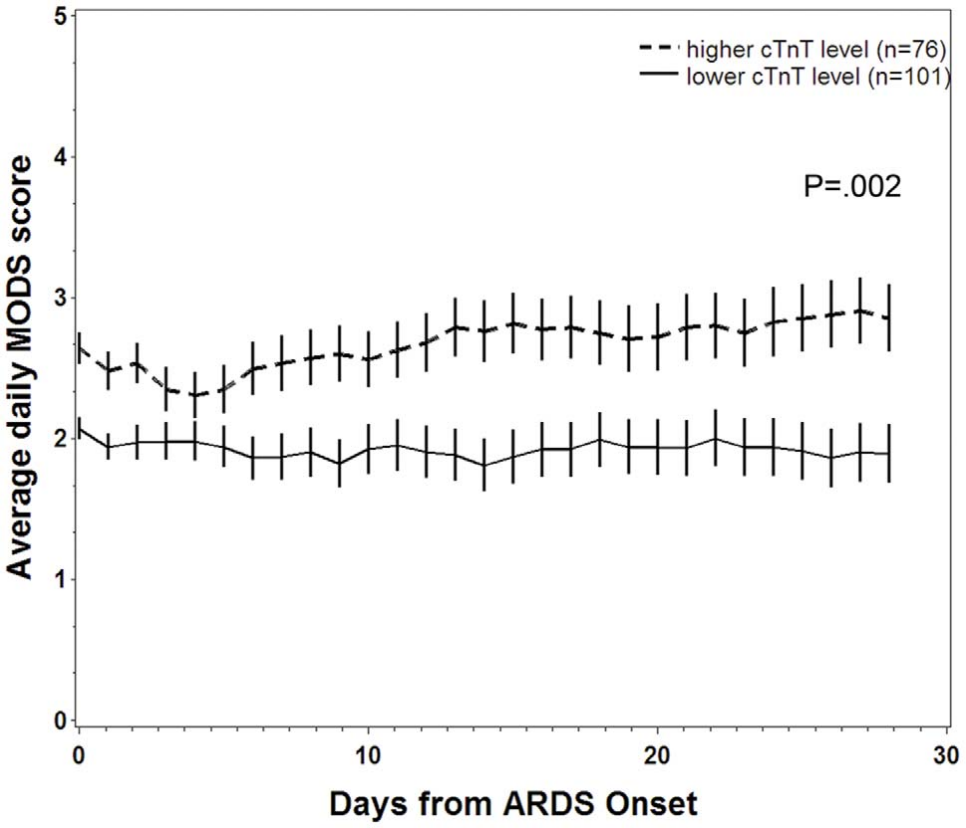
Daily MODS score by cTnT stratum. (Vertical bars indicate standard error of means, *P* value stated for repeated-measures ANOVA).

Patients with cTnT levels above the selected cutpoint had significantly higher daily MODS scores than those with normal cTnT levels, as shown in Figure 3 (median 2.5, IQR 1.2–4.1, vs. median 1.3, IQR 0.7–3.4, *P* = .002) and had significantly fewer ventilator-free days (median 0 days, IQR 0–9, vs. median 6 days, IQR 0–14, *P* = .03).

### Mortality Prediction

By itself, cTnT level had modest ability to predict mortality, with area under the ROC curve of (AUROC) 0.62 (95% CI 0.53–0.71), and sensitivity 57%, specificity 66%, negative predictive value (NPV) 70%, and positive predictive value (PPV) 52% at the optimal cut-point. By itself, APACHE III curve had an AUROC of 0.72 (95% CI 0.54–0.80) for predicting mortality, with sensitivity 64%, specificity 72%, NPV 75%, and PPV 60% at an APACHE III score of 84. The combined model of cTnT level, age, APACHE III score, and presence of acute hepatic failure improved predictive ability, with AUROC 0.81 (95% CI 0.75–0.87), sensitivity 63%, specificity 82%, NPV 77%, and PPV 70%.

### Echocardiographic/ECG Findings and cTnT

Eighty-one patients (46.3%) out of the study population had an echocardiogram obtained within 48 hours of cardiac biomarker blood draw. Fifty six patients (32%) had an available ECG within 48 hours of biomarker blood draw. Thirty three patients (19%) had both an echocardiogram and an ECG available, and sixty nine patients (39%) had neither. Because ECG and echocardiograms were obtained at the discretion of treating physicians, we sought to identify whether patient-specific factors might subject these data to selection bias. We compared these factors between the groups of patients who received echocardiogram and ECG testing and those who did not (table 3). We found that echocardiograms were obtained more frequently in patients with pneumonia or septic shock, but that otherwise they were similar to other patients with regard to age, severity of illness, cTnT level, diagnosis, and cardiac history. Patients who received ECG testing were also similar to those who did not, across all of the measured variables.

**Table 3:** Characteristics of patients who received echocardiogram or ECG testing.

	Echo not done (n = 96)	Echo done (n = 81)	*P*
Age (years)*	61.0 (45.0–77.0)	65.0 (49.0–74.0)	.74
APACHE III score	78.0 (65.0–97.0)	80.0 (70.0–96.0)	.51
cTnT level (ng/mL)	0.03 (0–0.09)	0.02 (0–0.1)	.90
***Female gender***	41 (44%)	36 (44%)	.96
Caucasian race	86 (92%)	76 (94%)	.73
Pneumonia	61 (66%)	68 (84%)	.006
Septic shock	48 (52%)	55 (68%)	.03
Sepsis (w/o shock)	32 (34%)	21 (26%)	.23
Trauma	5 (5%)	2 (2%)	.33
Aspiration	9 (10%)	6 (7%)	.59
Multiple transfusions	10 (11%)	4 (5%)	.16
Acute hepatic failure	4 (4%)	0 (0%)	.06
Known coronary artery disease	23 (24%)	21 (26%)	.82
Prior myocardial infarction	14 (15%)	12 (15%)	.99
Prior coronary artery bypass grafting	3 (3%)	7 (9%)	.12
Hyperlipidemia history	19 (20%)	14 (17%)	.62
Hypertension history	39 (42%)	35 (43%)	.82
Congestive heart failure	11 (12%)	7 (9%)	.51
	**ECG not done (n = 121)**	**ECG done (n = 56)**	***P***
Age (years)*	63.0 (45.0–74.0)	63.0 (47.0–77.0)	.45
APACHE III score	80.0 (66.0–99.0)	76.0 (68.0–95.0)	.48
cTnT level (ng/mL)	0.03 (0–0.08)	0.03 (0–0.12)	.60
***Female gender***	57 (48%)	20 (36%)	.15
Caucasian race	108 (91%)	54 (98%)	.07
Pneumonia	91 (76%)	38 (69%)	.30
Septic shock	65 (55%)	38 (69%)	.07
Sepsis (w/o shock)	41 (34%)	12 (22%)	.09
Trauma	4 (3%)	3 (5%)	.51
Aspiration	11 (9%)	4 (7%)	.67
Multiple transfusions	7 (6%)	7 (12%)	.12
Acute hepatic failure	3 (3%)	1 (2%)	.77
Known coronary artery disease	28 (24%)	16 (29%)	.47
Prior myocardial infarction	17 (14%)	9 (16%)	.76
Prior coronary artery bypass grafting	7 (6%)	3 (5%)	.89
Hyperlipidemia history	22 (18%)	11 (20%)	.86
Hypertension history	46 (39%)	28 (50%)	.16
Congestive heart failure	12 (10%)	6 (11%)	.90

Continuous variables are presented as median (interquartile range), categorical variables as number of patients in each category and (% of the total category).

Echocardiographic and electrocardiographic characteristics for the study population are shown in table 4. The median left ventricular ejection fraction was 57% (intraquartile range 23% to 80%). Nineteen patients (24%) had right ventricular dilation, and 18 (23%) had right ventricular hypokinesis. Sixteen patients (20%) had echocardiographic regional wall motion abnormalities. In three of these patients, this was a new finding compared to previous echocardiograms; in the remainder it was unchanged from prior studies, or no previous echocardiogram was available for comparison.

**Table 4:** Echocardiogram and electrocardiogram characteristics of study population.^a^

*Echocardiographic characteristics (n = 81)*	
Left ventricular ejection fraction (%)	57 (43–67)
***Left atrial size (mm)***	35 (30–40)
Left ventricular systolic size (mm)	32 (26–36)
Left ventricular diastolic size (mm)	44 (39–50)
Posterior wall thickness (mm)	10 (9–12)
Interventicular septal thickness (mm)	10 (8–12)
Left ventricular mass (grams)^b^	145.4 (112.5–191.3)
Right ventricular systolic pressure (mm Hg)	44 (38–53)
Mitral regurgitation grade^c^	1 (1–2)
Tricuspid regurgitation grade^c^	2 (1–2.5)
Regional wall motion abnormalities	16 (20%)
Right ventricular dilation	19 (24%)
Right hypokinesis	18 (23%)
***Electrocardiographic characteristics (n = 56)***	
Ventricularly paced	4 (7.1%)
ECG with any sign of ischemia	23 (41%)
ST elevation	1 (1.8%)
ST depression	5 (8.9%)
T wave inversion	10 (18%)
T wave flattening	8 (14%)
Left bundle branch block	2 (3.6%)

*^a^*For patients with a recorded echocardiogram or electrocardiogram within 48 hours of biomarker draw date. Continuous variables are presented as median (interquartile range), categorical variables as number of patients and (% of patients with available data).

*^b^*LV mass calculated using: Left ventricular mass (g) = 0.8 × 1.04 × [(Left ventricular end diastolic size + interventricular septal thickness + posterior wall thickness)^3^– left ventricular end diastolic size^3^] +0.6.

*^c^*Valvular regurgitation variables defined as follows: 0 = none, 1 = trace, 2 = mild, 3 = moderate, 4 = severe.

Tables 5 and 6, respectively, show echocardiogram and electrocardiogram characteristics for the study population, stratified by presence or absence of elevated cTnT. Patients with cTnT above the selected cut-point were significantly more likely to have echocardiographic evidence of regional wall motion abnormalities. Six out of the sixteen patients with echocardiographic wall motion abnormalities had prior echocardiograms available for comparison. Only three patients had wall motion abnormalities that were verifiably new compared to previous studies, and only two of these three were in the group with elevated cTnT. Patient with elevated cTnT had a higher median grade of tricuspid regurgitation (TR) than did patients with lower cTnT (P  = 0.04). There were no significant differences in ECG characteristics between the two groups, although there was a borderline significant association between presence of pathologic Q waves in patients with elevated cTnT vs. patients with normal cTnT (26% vs. 7%, P = 0.053). However, pathological Q waves were only noted in a total of 9 patients in the cohort. There was no relationship between the presence of ECG Q waves and echocardiographic regional wall motion abnormalities (*P* = .60).

**Table 5:** Echocardiogram characteristics, by cardiac marker status (n = 81).^a^

*Echocardiographic characteristics*	Positive troponin(TnT ≥0.03)	Negative troponin(TnT <0.03)	P**
Left ventricular ejection fraction (%)	57.5 (40–71)	56 (48–67)	0.87
***Left atrial size (mm)***	35.0 (31.5–40.5)	34.5 (29.0–39.0)	0.87
Left ventricular mass (grams)^b^	147.8 (113.6–186.6)	142.9 (100.6–205.5)	0.89
Right ventricular systolic pressure	44.5 (38–58)	42 (39–51)	0.55
Mitral regurgitation grade^c^	1 (1–2)	1 (0–2)	0.24
Tricuspid regurgitation grade^c^	2 (1.75–2.75)	2 (1–2)	0.04
Regional wall motion abnormalities	13 (37%)	3 (7%)	0.001
Right ventricular dilation	9 (25%)	10 (23%)	0.86
Right ventricular hypokinesis	9 (26%)	9 (21%)	0.62

*^a^*For patients with a recorded echocardiogram within 48 hours of biomarker draw date. Continuous variables are presented as median (interquartile range), categorical variables as number of patients and (% of patients with available data).

*^b^*For continuous variables, value represents P value for rank sum test for difference in distributions, for categorical variables, value represents P value for Chi2 statistic.

*^c^*Valvular regurgitation variables defined as follows: 0 = none, 1 = trace, 2 = mild, 3 = moderate, 4 = severe.

**Table 6:** Electrocardiogram characteristics, by cardiac marker status (n = 56).^a^

*Electrocardiographic characteristics*	Positive troponin(TnT ≥0.03)	Negative troponin(TnT <0.03)	*P*
ECG with any sign of ischemia	13 (48%)	10 (35%)	0.30
ST elevation	0 (0%)	1 (4%)	0.49
ST depression	4 (15%)	1 (4%)	0.14
Pathologic Q waves	7 (26%)	2 (7%)	0.05
T wave inversion	5 (19%)	5 (17%)	0.87
T wave flattening	6 (22%)	2 (7%)	0.10
Left bundle branch block	1 (4%)	1 (4%)	0.96

*^a^*Variables are presented as number of patients and (% of patients with available data).

Given the increased proportion of echocardiograms obtained in patients with septic shock or pneumonia, we sought to evaluate whether these results were confounded by the presence or absence of these diagnoses. We found no difference in the proportion of regional wall motion abnormalities present between patients with and without pneumonia (*P* = .23) or with and without septic shock (*P* = .74). Likewise, we found no differences between median TR grade between patients with and without pneumonia (*P* = .81) or those with and without septic shock (*P* = .11).

## Discussion

We have shown that cTnT levels are frequently elevated in patients with ARDS, and that cTnT elevation is associated with adverse outcomes, including death, organ failure, and need for mechanical ventilation. The association of increasing cTnT level with mortality from ARDS remains consistent after adjustment for other factors involved in outcome and is robust through a variety of statistical analyses. A multivariate model for mortality that includes cTnT shows a statistically stronger association than for the well-accepted APACHE III prognostic score. In addition, we have shown that there were few significant differences in electrocardiographic and echocardiographic parameters between patients with ARDS with and without elevated cTnT. In particular, it did not appear that patients with ARDS and elevated cTnT are having missed classical (or “type 1”) acute MIs or other variants of the acute coronary syndrome.

There are multiple mechanisms whereby myocardial necrosis may occur in ARDS patients. It is plausible that the pathogenesis is akin to that of myocardial injury sustained by patients with severe sepsis/septic shock [21]. However, these results show that many ARDS patients had detectable cTnT levels even in the absence of suspected infection. Moreover, plasma cTnT had independent value in predicting mortality when compared with the myriad clinical data represented by the APACHE III score. This suggests that myocardial necrosis could be important in the pathogenesis of ARDS beyond being a hallmark of systemic disease such as septic shock. However, some of the molecular mechanisms postulated to be involved in septic myocardial dysfunction may also be in play in ARDS even in the absence of infection or shock, including circulating proinflammatory mediators, nitric oxide activity, matrix metalloproteinase activation, mitogen-activated protein kinase activity, and induction of cellular apoptosis [21].

Another possible explanation for these findings is that flow-limiting atherosclerotic lesions due to CHD may predispose individuals to ischemic myocardial necrosis in the setting of increasing myocardial oxygen demand and reduced coronary blood flow due to critical illness (so-called type II MI). Supporting this explanation are the findings of more regional wall motion abnormalities and a trend toward more pathologic Q waves in the group with elevated cTnT, which may indicate a greater penetrance of CHD and flow-limiting coronary lesions in that group. Contradicting this explanation, however, are data that coronary blood flow is increased, not decreased, in patients with septic shock, and that elevated troponin levels in a majority of critically ill patients were not associated with flow-limiting atherosclerotic coronary artery lesions in a prior study [22], [23]. Furthermore, we did not observe significant differences between patients with and without cTnT elevations with respect to any ECG signs of active myocardial ischemia or acute coronary syndromes. While patients with elevated cTnT were more likely to have regional wall motion abnormalities on echocardiography, which can be present in both active cardiac ischemia and in patients with a history of myocardial infarction, this finding is not specific to CHD and was seen in a minority of patients. It is possible that patients with ARDS may be at risk of demand-related myocardial ischemia independent of CHD. In this scenario, cardiac biomarker elevation from such type II myocardial infarctions may not be associated with classic electrocardiographic signs of ischemia or specific findings on echocardiography. Further research is needed in patients with ARDS to investigate both the significance of regional wall motion abnormalities and the possibility of demand-related myocardial ischemia in the setting of biomarker evidence of myocardial necrosis.

Pulmonary vascular disease and associated right ventricular strain are likely contributors to myocardial necrosis in ARDS. In particular, pulmonary vascular disease is known to accompany ARDS, and hemodynamic evidence of right ventricular strain has been implicated in poorer prognosis [8], [24]. Other reports have shown that increased pulmonary dead space and radiologically-determined pulmonary vascular obstruction are associated with worse outcomes in ARDS [25], [26]. Surprisingly, our data show that patients with elevated cardiac biomarkers are no more likely to show right ventricular hypokinesis, right ventricular dilation, or elevated right ventricular systolic pressure on transthoracic echocardiography, than are patients with normal levels of cTnT. The validity of our data is supported by our finding that between 22 and 33% of patients had evidence of right ventricular dilation or hypokinesis, which is very similar to previously reported rates of echocardiographically demonstrated acute cor pulmonale in patients with ARDS [27]. There are other potential explanations for the lack of correlation between cTnT and RV dysfunction. First, pulmonary vascular disease in ARDS may be predominantly microvascular and relatively sub-acute in onset, and thus obtaining an echocardiogram within 48 hours of ARDS onset may be too early for echocardiographic signs of right ventricular strain to manifest differently in patients with and without biomarker evidence of myocardial necrosis. Secondly, right ventricular strain occurring in patients with ARDS may not be effectively captured by a echocardiographic assessments of right ventricular hypokinesis or systolic function, even if a substantial degree of strain is present. Previous reports have shown that compared to magnetic resonance imaging, echocardiographic reliability for accurately identifying moderately to severely diminished right ventricular function was slight to fair, with poor inter-rater agreement [28]. There are abundant data that echocardiographically obtained pulmonary artery and right ventricular systolic pressures are inaccurate, and do not correlate well with measurements made during gold standard right heart catheterization, even in patients with known pulmonary hypertension [29]–[31]. Conversely, echocardiographic determination of the presence and severity of tricuspid regurgitation is highly accurate, and correlates well with assessments made by right ventriculography [32], [33]. In our study participants, there was a significantly greater median grade tricuspid regurgitation in patients with elevated cTnT than inpatients with negative biomarkers. Given the high reliability of echocardiographic determinations of triscuspid regurgitation, this may represent a meaningful difference in right sided heart function between the two groups that is not evident from other echocardiographic measurements. Previous reports have demonstrated that tricuspid regurgitation is a common finding in the critical care setting in conditions known to be characterized by right ventricular strain, such as acute pulmonary embolism [34]. A final explanation for the discrepancy between cTnT and echocardiographic evidence of RV dysfunction is that right ventricular strain with myocardial necrosis my be common in patients with ARDS, and may be independent of disease severity as marked by elevated cTnT.

The strengths of this study include its prospective nature and well-characterized population. Use of NAECC criteria to define ARDS limits misclassification bias related to the lack of a diagnostic gold-standard. We dealt with bias inherent to the prior study by including only cTnT assays in our study and blinding investigators to the clinical status of patients. This is a potential reason that we did not find evidence of the previously suggested effect modification by age or severity of illness. In the prior study, the decision to test for biomarkers of myocardial necrosis was made by unblinded clinicians; patients who had blood tested for biomarker levels were significantly more likely to be older and to have higher severity of illness scores than those who were not tested. In addition, the analysis of available echocardiographic and ECG data for a portion of study patients allowed us to evaluate for the presence of preexisting or concomitant CHD and ACS, which may obviously influence biomarker levels in this clinical setting.

Historically, much attention has been paid to using physiologic measurements to assess for severity of illness in ARDS. However, such measurements may be cumbersome to perform, require extensive training, or pose substantial risks to patients. Recent research has focused on the definition of molecular phenotypes using biomarker analysis. This has included attempts to detect or quantify various aspects of ARDS pathophysiology, including systemic inflammation, alveolar-capillary leak, and altered coagulation [35]–[38]. Thus far these efforts have ranged from highly targeted approaches that study either single or small groups of biomarkers, to more expansive and ambitious efforts that attempt to analyze the entire proteome of such patients [39]. Our approach shows that biomarkers of myocardial necrosis such as cTnT may comprise an important part of attempts to further define the molecular phenotype of ARDS.

We acknowledge the presence of several limitations of our study. As a general limitation, the design of this study makes it difficult to generalize the results to other populations of patients, including the immunocompromised or patients with risk factors for ARDS other than those studied. In terms of the data available, echocardiographic and ECG results were only available for a portion of the study patients, as the decision to obtain these studies was made by treating physicians in the process of routine clinical care. Therefore these data are limited by absence of data for all study patients, thus introducing potential selection bias. To address this issue, we compared characteristics of patients who received echocardiogram/ECG and those who did not, and found few differences between these groups. We did find that pneumonia or septic shock were more frequently present in patients who had echocardiograms, but there was no evidence of confounding between these initial diagnoses and the presence of the reported echocardiographic abnormalities. In addition, because of the small numbers of echocardiograms and electrocardiograms available for analysis, the power of these analyses to detect small differences is limited. A further limitation was our inability to capture serial echocardiograms over time for study participants. Zapol et. al demonstrated that in patients with severe acute respiratory failure, initial levels of pulmonary artery pressure or pulmonary vascular resistance were not prognostic [8]. However, the failure of pulmonary artery pressure and pulmonary vascular resistance to fall over time was associated with decreased survival. It is therefore possible that had we obtained serial echocardiograms for our study participants, we may have found a correlation between biomarker evidence of myocardial necrosis and echocardiographic measures of right ventricular or pulmonary artery hemodynamics. For similar reasons, it would have been ideal to capture serial measurements of cTnT to determine whether the relationship between biomarker level, cardiac function, and prognosis vary with serial measurement. Future studies should continue to further explore troponin elevation with regard to cardiac function in hopes of clarifying their relationship, and we hope this study stimulates interest in such research. In particular, new-generation high-sensitivity troponin assays have recently become available [40]. These assays can detect troponin in the blood at levels considerably lower than can be measured by conventional assays. Since troponin levels in this population tended to be in the lower ranges of the assay, high-sensitivity assays might improve predictive ability and should be examined in future studies.

### Conclusions

We have shown that biomarker evidence of myocardial injury is associated with worsened outcomes in patients with ARDS, and that this appears to be independent of other systemic factors such as severity of illness or the presence of infection. In addition, elevated cardiac-specific biomarkers in this population are associated with regional wall motion abnormalities and with triscuspid valve dysfunction by echocardiography, but are not associated with ECG evidence of acute coronary syndrome. This research raises the potential importance of cardiac function in ARDS and contributes to efforts to establish define molecular phenotypes in this syndrome. Further investigation is warranted to define these relationships and apply the information garnered to the tasks of diagnosis, management, and prognostication.
